# Endometriosis and Phytoestrogens: Friends or Foes? A Systematic Review

**DOI:** 10.3390/nu13082532

**Published:** 2021-07-24

**Authors:** Ludovica Bartiromo, Matteo Schimberni, Roberta Villanacci, Jessica Ottolina, Carolina Dolci, Noemi Salmeri, Paola Viganò, Massimo Candiani

**Affiliations:** 1 Gynecology/Obstetrics Unit, IRCCS San Raffaele Scientific Institute, 20132 Milan, Italy; bartiromo.ludovica@hsr.it (L.B.); schimberni.matteo@hsr.it (M.S.); villanacci.roberta@hsr.it (R.V.); ottolina.jessica@hsr.it (J.O.); dolci.carolina@hsr.it (C.D.); salmeri.noemi@hsr.it (N.S.); candiani.massimo@hsr.it (M.C.); 2 Fondazione IRCCS Ca’ Granda Ospedale Maggiore Policlinico, 20122 Milan, Italy

**Keywords:** phytoestrogens, endometriosis, lignan, resveratrol, flavonoid

## Abstract

The aim of this systematic review was to provide comprehensive and available data on the possible role of phytoestrogens (PE) for the treatment of endometriosis. We conducted an advanced, systematic search of online medical databases PubMed and Medline. Only full-length manuscripts written in English up to September 2020 were considered. A total of 60 studies were included in the systematic review. According to in vitro findings, 19 out of 22 studies reported the ability of PE in inducing anti-proliferative, anti-inflammatory and proapoptotic effects on cultured cells. Various mechanisms have been proposed to explain this in vitro action including the alteration of cell cycle proteins, the activation/inactivation of regulatory pathways, and modification of radical oxidative species levels. Thirty-eight articles on the effects of phytoestrogens on the development of endometriotic lesions in in vivo experimental animal models of endometriosis have been included. In line with in vitro findings, results also derived from animal models of endometriosis generally supported a beneficial effect of the compounds in reducing lesion growth and development. Finally, only seven studies investigated the effects of phytoestrogens intake on endometriosis in humans. The huge amount of in vitro and in vivo animal findings did not correspond to a consistent literature in the women affected. Therefore, whether the experimental findings can be translated in women is currently unknown.

## 1. Introduction

Endometriosis is a common benign chronic disease affecting reproductive-age women [1]. It is defined as the presence of endometrial tissue and fibrosis located outside the uterus and is frequently associated with pelvic pain, infertility, urinary and bowel dysfunction [2,3,4]. As a hormonal disease characterized by features of a chronic inflammatory condition, various theories on its development based on an uncontrolled hormonal response and immune-mediated dysfunctions have been proposed [5,6]. Estrogens are key promoters of endometrial cellular growth. Any insult that affects estradiol biosynthesis and catabolism in women with endometriosis have been proposed to play a part in aberrant cell growth. Levels of peripheral estrogens do not seem however altered in women with endometriosis. On the other hand, estrogens are both endocrine and paracrine agents and one may speculate that even a modest variation of estrogen production may be somehow detrimental locally. Indeed, locally accumulated estradiol can create an estrogenic microenvironment around endometriotic lesions. High local concentrations of estradiol and alterations in estrogen receptor (ER) α and ERβ receptor expression may activate a network of genes regulating cell proliferation [7,8]. In line with these observations, medical treatment for endometriosis is still focused on pain and lesion size control with hormonal therapies able to establish either a hypo-estrogenic or a hyper-progestogenic milieu [9,10]. In this context, a role of diet has been postulated based on the idea that estrogen activity can be influenced by nutrition [11,12]. In other conditions in which hormones exert a specific role, such as breast and endometrial carcinogenesis, research has demonstrated that diet may strongly affect incidence [13].

Phytoestrogens (PE) have been identified in various types of food stuffs including fruits, vegetables, sprouts, beans, cabbage, soybean, grains, tea and oilseeds. Based on their structure, the main classes of PE consist of flavonoids (i.e., puerarin, genistein, coumestrol, epicatechin and naringenin), lignans (i.e., enterolactone), and stilbenes (i.e., resveratrol). Classified into three main classes, PE include flavonoids (i.e., puerarin, genistein, coumestrol, epigallocatechin gallate (EGCG), naringenin, quercetin), lignans (i.e., eneterolactone), and stilbenes (i.e., resveratrol). [14]. Their close structural similarity to estrogens, characterized by a phenolic ring and two hydroxyl groups, allows them to act as weak estrogenic factors and to interfere with hormonal and molecular signaling, having positive effects including the prevention of menopausal symptoms, type 2 diabetes, cardiovascular disease, obesity and cancer [15]. Moreover, PE may have poor estrogenic activity in low-estrogen environments such as in menopause and have antiestrogenic activity in high-estrogen environments such as those observed in endometriosis or endometrial cancer [16,17]. Several studies have evaluated the associations between PE and endometriosis risk in animal and human models but the data obtained are quite inconsistent or conflicting [18,19,20,21,22,23,24,25,26,27,28,29,30,31,32,33,34,35,36,37,38,39,40,41,42,43,44,45,46,47,48,49,50,51,52,53,54,55,56,57,58,59,60,61,62,63,64,65,66,67,68,69,70,71,72,73,74,75,76,77].

The aim of this systematic review was to gain insight into the mechanisms of action of PE in endometriosis and to offer a general view of available data on their possible role for the treatment of endometriosis.

## 2. Materials and Methods

The study protocol was registered “a priori” and accepted for inclusion in PROSPERO (PROSPERO ID CRD42020220847). The methods for this systematic review were developed in accordance with the Preferred Reporting Item for Systematic Reviews and Meta-analysis (PRISMA) guidelines [78]. No Institutional Review Board Approval was needed. We performed an advanced, systematic search of online medical databases PubMed and Medline using the following keywords: “endometriosis” in combination with “phytoestrogen”, “flavonoid”, “non-flavonoid”, “isoflavone”, “coumestan”, “lignan” and “resveratrol”. To optimize search output, we used specific tools available in each database, such as Medical Subject Headings (MeSH) terms (PubMed/Medline). The EndNote software (available online: https://endnote.com, accessed on 19 September 2020) was used to remove duplicate articles. Only full-length manuscripts written in English up to September 2020 were considered. We checked all citations found by title and abstract to establish the eligibility of the source and obtained the full text of eligible articles. We also performed a manual scan of the references list of the review articles to identify any additional relevant citations. Three review authors (R.V., M.S. and L.B.) independently assessed the risk of bias for each study using the risk-of-bias tool for case–control studies developed by clarity group [79]. We assessed the risk of bias according to the following domains: (i) Can we be confident in the assessment of exposure?; (ii) Can we be confident that cases had developed the outcome of interest and controls had not?; (iii) Were the cases properly selected?; (iv) Were the controls properly selected?; (v) Were cases and controls matched according to important prognostic variables or was statistical adjustment carried out for those variables? We graded each potential source of bias as Definitely yes (low risk of bias), Probably yes (Moderate risk of bias), Probably no (Serious risk of bias), or Definitely no (Critical, high risk of bias). We summarized the risk of bias judgments across different studies for each of the domains listed.

## 3. Results

**Figure 1:**
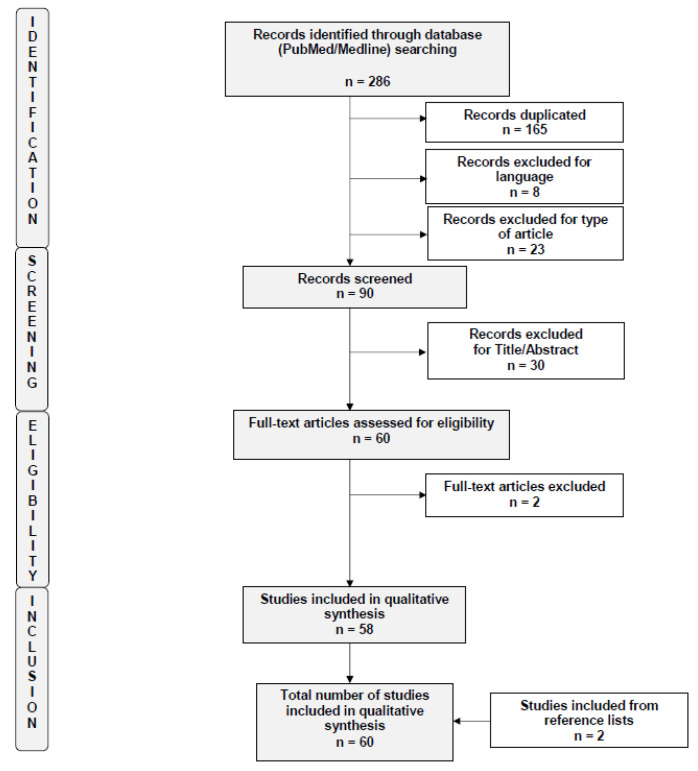
Flow diagram of the search strategy, screening, eligibility and inclusion criteria.

A total of 286 studies were initially identified by the search criteria. After applying the selection criteria, a total of 60 trials were included in the systematic review [18,19,20,21,22,23,24,25,26,27,28,29,30,31,32,33,34,35,36,37,38,39,40,41,42,43,44,45,46,47,48,49,50,51,52,53,54,55,56,57,58,59,60,61,62,63,64,65,66,67,68,69,70,71,72,73,74,75,76,77]. A flow diagram of the systematic review is shown in Figure 1 (PRISMA template). The risks of bias of the included studies are summarized in Supplementary Figure S1. Findings derived from the studies are herein presented based on in vitro results, evidence in in vivo animal models and finally in humans.

### 3.1. Studies Included

#### 3.1.1. Phytoestrogens and Endometriosis: In Vitro Experimental Human Models

Several studies tried to assess PE effect on human endometrial/endometriotic cells. The results from 22 studies are summarized in Table 1. What is surprising is the heterogeneity of the substances studied and their respective biological effects, although for some of them it is possible to designate common actions.

Resveratrol was the most studied substance whose effects have been investigated in 5 out of 22 studies with significant findings in all of them [35,41,47,59,74]. According to Ricci and coworkers [35], resveratrol could induce significant changes in cell proliferation and apoptosis of eutopic endometrial epithelial cell cultures although without significant differences between endometriosis patients and control women. In line, Khazei et al. have recently reported that the anti-proliferative, proapoptotic and anti-angiogenetic effect of this substance was not specific for ectopic endometrial cells [74].

They claimed a role for the treatment in reducing nitric oxide (NO) levels, found to be higher in endometriotic cells, and in increasing significantly the expression of apoptotic genes (P53, Bax, Bcl2 and caspase 3) and of sirtuin 1 (SIRT1) in both eutopic and ectopic cells. A relationship between activation of SIRT1 by resveratrol and interleukin (IL)-8 was investigated by Taguchi et al. [41] who, conversely, demonstrated that the anti-inflammatory effects of the compound were more prominent in endometriotic cells than in eutopic cells from controls. The same group, one year later, reported that, even if resveratrol alone was not capable of inducing apoptosis in endometriotic cells, it determined an altered expression of some key molecules involved in apoptosis such as survivin or TNF-α-related-apoptosis-inducing ligand (TRAIL), favoring cell death in ectopic lesions [47]. Finally, a higher insulin-like growth factor-1 (IGF-1) and hepatocyte growth factor (HGF) gene expression in ectopic endometrial cells has been demonstrated by Arablou and coworkers [59]. In this case, resveratrol biological effect in terms of decrease in IGF-1 and HGF protein production was reported for both eutopic and ectopic endometrial stromal cells from women with endometriosis but not for cells from controls. Resveratrol was also shown to inhibit IGF-1/ERK and HGF/MAPK signal transduction pathways in a dose-dependent manner, thus resulting in anti-inflammatory and anti-proliferative effects. Therefore, although the exact mechanism involved is still poorly defined, all the papers supported some in vitro benefit of resveratrol.

Three studies investigated the effects of puerarin (10^−9^ M), a major isoflavonoid compound extracted from the Chinese medicinal herb, *Radix puerariae* [28,30,34]. Studies were concordant in demonstrating that puerarin treatment in combination with ethinylestradiol (E2) significantly suppressed the E2-mediated proliferation of stromal cells from endometriotic lesions. Moreover, treating ectopic stromal cells with Puerarin abrogated ERK phosphorylation through a competition with estrogen for the binding to membrane receptors of MAPK signaling, thus significantly decreasing cell proliferation, as well as gene expression levels of cyclin D1, cyclo-oxygenase (COX) 2 and cyp19 involved in this process [30,34]. Finally, Ji and coworkers demonstrated that puerarin can partly suppress estrogen-stimulated proliferation by promoting the recruitment of corepressors to estrogen receptor, as well as limiting that of coactivators, in order to arrest ectopic stromal cells in the G1 phase [34]. Three studies out of 22 investigated the biological effect of chyrisin, a natural compound derived from honey, propolis, or passion flowers, on human endometrial cells [20,66,75]. Although shown to be potent inhibitor of aromatase activity in a free cell assay, chyrisin, daidzein or naringenin could not attenuate aromatase activity in endometrial stromal cells in women with and without endometriosis at any concentration tested. Only genistein (10^−9^–10^−6^ M) indirectly increased aromatase activity in endometrial stromal cells from controls. On the other hand, in both VK2/E6E7 and End1/E6E7 endometriotic cell lines, chyrisin was shown to suppress cell proliferation and induced the programmed cell death through changing the cell cycle proportion, increasing the cytosolic calcium level and generating reactive oxygen species (ROS) [66]. In addition, Chrysin activated endoplasmic reticulum (ER) stress by stimulating the unfolded protein response proteins, especially the 78-kDa glucose-regulated protein, GRP78, the PRKR-like ER kinase (PERK) and the eukaryotic translation initiation factor 2α (eIF2α). Finally, the compound was shown to inactivate the intracellular phosphatidylinositol 3-kinase (PI3K)/protein kinase B signaling pathway in a dose-dependent manner from 5 to 100 μM. Similar results and the same biological mechanisms were reported for chyrisin by Park et al. [75], actually testing 5,7-Dimethoxyflavone (DMF), a methylated form of chrysin extracted from *Kaempferia parviflora* (KP). The methylation of flavonoids has been demonstrated to greatly increase their absorption and bioavailability. A similar biological effect was demonstrated for naringenin by the same authors [50]. Indeed, naringenin (100 μM) decreased the proliferation and increased apoptosis of VK2/E6E7 and End1/E6E7 cells. In the same cells, it also increased the production of ROS 3-fold, induced mitochondrial pro-apoptotic proteins (Bax and Bak), in VK2/E6E7 cells by ~7-fold and in End1/E6E7 cells by 2-fold. Finally, naringenin significantly increased apoptosis through generation of ER stress regulatory genes, in particular G1 arrest and DNA damage 153 (GADD153), inositol-requiring protein 1α (IRE1α) and GRP78, and through activation of MAPK signaling and inactivation of PI3K pathway. It is interesting to note that the same group of authors investigated these same biological mechanisms highlighted for chirisin, narigenin for other substances, such as apigenin, delphinidin, luteolin, quercetin, silibinin and myricetin in VK2/E6E7 and End1/E6E7 endometriotic cells lines [50,55,61,67,68,69]. All these studies are summarized in Table 1. Overall, they demonstrated that the PE effect on endometriosis is always antiproliferative and proapoptic through the activation of intracellular signals of calcium, ER stress and ROS production and through the activation of the MAPK pathway and a decreased phosphorylation of ERK1/2 and PI3K/AKT signaling proteins.

Two studies out of 22 investigated the biological effect of EGCG in eutopic endometrial stromal cells (EuSC) from women with or without endometriosis [35] or in EuSC and ectopic endometrial stromal cells (EcSC) from women affected by endometriosis [40]. The results from these studies were contradictory: while Ricci and coworkers showed no significant difference in cell proliferation and apoptosis between cases and controls [35], Matsuzaki et al. demonstrated an inhibited cell proliferation, migration and invasion of both EuSC and EcSC after EGCG treatment. Moreover, EGCG significantly decreased the Tumor growth factor b-1 (TGF-b1)-dependent increase in the mRNA expression of fibrotic markers and significantly inhibited TGF-b1-stimulated activation of the MAPK and Smad signaling pathways in both cells [40].

Kim et al. [48] examined the effect of Pueraria flowers extract (PFE), a rich source of isoflavones such as genistein, daidzein, kakkalide, puerarin, and tectoridin, on immortalised human endometriotic cells, 11Z and 12Z. Mesothelial Met5A cells were used for adhesion assessment after PFE treatment. They concluded that PFE significantly inhibited adhesion and migration of endometriotic cells to mesothelial cells, suppressing the mRNA and protein expressions of matrix metalloproteinases (MMP)-2 and MMP-9 and increasing the phosphorylation of ERK1/2 in endometriotic cells. A decreased MMP expression was also reported for apigenin [55] and for quercetin [68].

Takaoka and coworkers showed that Daidzein-rich isoflavone aglycones (DRIAs) significantly inhibited the proliferation of ectopic cells in a concentration dependent manner [57]. It also decreased IL-6, IL-8, COX-2 and aromatase mRNA levels, prostaglandin E2 (PGE2) protein levels, and aromatase enzyme activity. DRIAs suppressed the Tumor necrosis factor-α (TNF-α) induced IκB expression, the nuclear factor-κB-IKB-inhibitory proteins (NF-kB-ikb) complex formation, and the uptake of p65 into the nucleus.

In contrast to all this evidence, Hernandes and colleagues have shown that rutin, a glycosylated flavonoid and extract of *Uncaria guianensis,* or a combination of both, was not able to reduce cellular viability, although ROS production did increase in both eutopic and ectopic cells [71]. In addition, significant increases levels of interleukin (IL)-15, IL-17A, IL-4, IL-6, TNF-α, and vascular endothelium growth factor (VEGF) were observed when eutopic endometrial cells were treated with aqueous bark extract of *U. guianensis* (ABE), while exposure to aqueous bark leaf of *U. guianensis* (ALE) induced significant increases in epidermal growth factor in lesion cells.

#### 3.1.2. Phytoestrogens and Endometriosis: In Vivo Experimental Animal Models

Thirty-eight articles on the effects of PE on the development of endometriotic lesions in in vivo experimental animal models of endometriosis have been included in this systematic review. Among them, PE were administered non orally (18 studies, Table 2), orally (18 studies, Table 3) or both (2 studies, reported in both tables). Seven studies investigated the effects of EGCG on endometriosis development, five in mice and one in hamsters. ECGC was administered either orally [35] or through an intraperitoneal injection [23,24,29,35,37,40,45]. In all the studies in which endometriotic lesions were measured, ECGC induced the regression of lesions.

The compound could suppress E2-stimulated activation, proliferation and VEGF expression of endometrial cells in vitro isolated from hamsters. When evaluated by intravital fluorescence microscopy and histology, in vivo treatment with ECGC mediated a selective inhibition of angiogenesis and blood perfusion of endometriotic lesions without affecting blood vessel development in ovarian follicles [23,24,35,37]. Moreover, EGCG showed to increase total apoptotic cell numbers in the lesions [24,35,37]. Molecular mechanisms put forward to explain these phenomena in the lesions include: selective inhibition of VEGFC expression; down-regulation of MMP-9, chemokine (C-X-C motif) ligand 3 (CXCL3), c-JUN, and interferon-γ expression, decreased ROS generation and lipid peroxidation and reduced MMP-2 and MMP-9 activity [29,45]. Matsuzaki and colleagues observed significantly lower scores for both Sirius red and Masson trichrome staining in EGCG-treated mice suggesting that this treatment may prevent the progression of fibrosis [40].

Eight studies investigated the effects of resveratrol on endometriosis development, three in rats and five in mice. Resveratrol was administered either orally [26,36,44], or through an intramuscular [33,43], subcutaneous [38] or intraperitoneal [35,42] injection. The administration of resveratrol showed a marked reduction in endometriotic implants when they were measured. Histological evaluations of tissue sections revealed that both the dimensions and the vascularization of the implants were diminished in the resveratrol-treated animal model. Molecular mechanisms proposed to explain this finding include: decreased levels of VEGF, monocyte chemotactic protein 1 (MCP-1), IL-6, IL-8 and TNF-α in the peritoneal fluid and lower presence of lesional MMP-2, MMP-9 and VEGF [33,35,36,43,44]. Investigating the effects of resveratrol on the expression of estrogen receptor α (ER-α), the proliferative marker Ki-67, aryl hydrocarbon receptor (AhR) and members of the cytochrome P450 superfamily of enzymes, Amaya and colleagues found that mice treated with estradiol (E2) plus progesterone or E2 plus the highest dose (60 mg) of resveratrol exhibited a reduction in both ER-α and Ki-67 in eutopic endometrial epithelial cells. In stromal cells, ER-α levels were reduced by E2 plus P, but not by resveratrol while Ki-67 expression was reduced in presence of 60 mg/day of resveratrol suggesting the potential benefit of high doses of the compound in reducing the proliferation of human endometrium [38]. Bruner-Tran and colleagues have demonstrated that oral administration of resveratrol at dose of 6 mg either for 10–12 days or 18–20 days decreased number of endometrial implants per mouse by 60% and the total volume of lesions per mouse by 80% [26]. Moreover, the authors studied the effect of resveratrol on EcSC in vitro invasiveness, finding a concentration-dependent reduction up to 78%. Finally, Yavuz and coworkers demonstrated a reduced oxidative stress in cases compared to controls in a dose-dependent manner (I.P injection of resveratrol at low dose 1 mg/kg and high dose 10 mg/kg), confirming also reduced lesion size and reduced proliferative scores for the treatment group independent of the dose [42].

The potential role for the genistein to sustain endometriosis has been explored by Cotroneo et al. and Laschke et al. [19,25]. Totally in disagreement with the other studies in this context, the subcutaneous and intraperitoneal injections of genistein was shown to sustain the growth of the implanted tissue in a dose-responsive manner [19] and not to sustain the neoangiogenesis and blood perfusion of endometriotic lesions [25]. When measuring uterine receptor expression, the treatment resulted in a significantly uterine decreased expression of ER-α protein and in an increased progesterone receptor (PR) expression at all doses compared to controls [19]. When administered orally, the same group of authors found that genistein determined an increase of uterine PR type B (PRB) at higher dietary dose. By contrast, in his previous research Yavuz et al. demonstrated that administered orally genistein resulted in smaller areas of endometriotic lesions and lower histological scores if compared with control animals [22].

Subcutaneous administration of silymarin [52] and intraperitoneal injection of silibinin, scutellarin, nobiletin, quercetin and myricetin have all been shown to reduce lesion size in mice and rats [58,60,61,68,76]. Ham et al. also found that the expression of TNF-α, IL-1β, and IL-6 mRNA decreased to 80.4%, 73.8%, and 96.5% respectively in the endometriotic lesions upon intraperitoneal silibinin treatment in mice [61]. Since scutellarin is traditionally used as a potent antiplatelet agent, Ding et al. evaluated its potential therapeutic effect showing also improved hyperalgesia in both low-dose and high-dose and changes consistent with reduced proliferation, angiogenesis, and fibrogenesis of the lesions. Moreover, this flavonoid also significantly reduced the platelet activation rate in peripheral blood when administered intraperitoneally in mice [60]. Intraperitoneal-injected nobiletin was shown to be effective on the activation of NF-κB in endometriotic cells, mainly targeting on the activity of IκB kinases (IKKs) and reducing p65 phosphorylation level [58]. A potential anti-proliferative role on endometriosis through cell cycle regulation has been demonstrated by Park et al. upon intraperitoneal administration of myricetin, quercetinin or luteolin in a mouse model [68,69,76].

In a rat model of endometriosis, oral administration of Puerarin inhibited the growth of ectopic implants and reduced estrogens levels in endometriotic tissue even when administered at low dose and without systemic adverse effects [27].

The potential therapeutic action of Xanthohumol, a flavonoid belonging to the same family of resveratrol, has been investigated by Rudzitis [32]. Similarly to resveratrol, oral Xanthohumol was able to reduce lesion growth by decreasing cell proliferation. Similar results were obtained with the oral administration of Sylimarin, Naringenin and Wogonin, plant-derived flavonoids [51,54,64]. Melekoglu et al. have evaluated the effect of hesperidin, a flavanone glycoside found in citrus fruit, on endometriosis development in a rat model observing lower lesion volumes and increased levels of antioxidant parameters when administered orally at dose of 50 mg/kg for 14 days [53].

Oral isoliquiriteginin (ISL), a flavonoid found in liquorice, has been found not only to decrease lesion volume but also to reduce serum and tissue VEGF, IL1β and IL-6 and to increase Bax, Bcl-2 and E-cadherin [72]. Other authors have investigated the effect of a daidzein-rich isoflavone aglycones diet [57] or of extract of different plants known to contain several PE such as *Achileea bierbersteinii* [39], *Urtica dioica* and *Anthemis austriaca* [62,63], *Melilotus officinalis* [73] and *Achillea critica* [70] on endometriosis lesions. All of them have been found to decrease the volume of lesion and adhesion scores. Also, decreased concentration TNF-α were observed both in peritoneal [62,63] and in serum and tissue samples [70]. Moreover, *Urtica dioica, Anthemis austriaca, Melilotus officinalis* and *Achillea critica* (AC) extract were able to reduce peritoneal VEGF and IL-6 compared to controls. The anti-inflammatory properties of AC were observed in the ability to reduce serum TNF-α, VEGF and IL-6 as well.

#### 3.1.3. Phytoestrogen Dietary Intake and the Risk of Endometriosis in Humans

Table 4 shows the results of the seven studies that have investigated the effects of PE intake on endometriosis in humans. The first study that evaluated the effects of intaking soy products such as genistein and daidzein found an inverse association between the isoflavone intake and the risk of undergoing premenopausal hysterectomy for benign gynecological conditions, including endometriosis [18]. Similar results have been obtained in a case-control study evaluating urinary levels of genistein and daidzein in 138 women. Levels of isoflavones were found to be inversely correlated to stage III-IV of the disease. Frequency of ER-2 gene RsaI polymorphism was also assessed. A significant association was noted between specific genotypes of ER-2 RsaI polymorphism and genistein levels in risk of advanced endometriosis. Since altered estrogen or soy isoflavone signal transduction thanks to ER-2 gene polymorphisms may be directly responsible for susceptibility to severe endometriosis, the authors suggested that isoflavones may play a more effective role among the ER-2 RsaI R/r + R/R genotype than the r/r genotype, although the latter itself is likely to be protective for endometriosis [21]. Three studies have evaluated the effects of resveratrol on endometriosis women [31,49,65].

## 4. Discussion

Most of the available therapies for endometriosis are hormonal-based therapies able to establish either a hypo-estrogenic or a hyper-progestogenic milieu [80,81,82]. Phytoestrogens are a heterogeneous group of naturally occurring compounds in plants structurally similar to estrogens [15]. They are characterized by a phenolic ring, which determines their agonist or antagonist properties, and two hydroxyl groups which are crucial for the binding to ER [15]. Classified into three main classes, PE include flavonoids (i.e., puerarin, genistein, coumestrol, EGCG, naringenin, quercetin), lignans (i.e., eneterolactone), and stilbenes (i.e., resveratrol) [14,83]. Flavonoids are characterized by a typical structure C6–C3–C6 with two rings of benzene A and B linked by a chain of 3 carbons cycled through an atom of oxygen [84]. Based on the connection, the position, the degree of saturation, oxidation, and hydroxylation of the B and C rings, they are commonly divided into isoflavones and coumestans [15,84,85,86]. Genistein and daidzein (up to 90% of isoflavones) are present in soybeans [87]. Among coumestans, coumestrol is one of the most studied and considered as an endocrine disruptor due to the high affinity in binding ERs [88], with an estrogenic activity greater than that of other isoflavones due to the position of its two hydroxyl groups [89]. It is present in a variety of plants including soybeans, clover, alfalfa sprouts, sunflower seeds, spinach, and legumes. Flavones, a subgroup of flavonoids whose main compound is apigenin, are characterized by a double bond between C2 and C3 that may induce cell cycle arrest and DNA damage in some cell types [90,91]. The skeleton and the position of phenolic group are the main characteristics of another flavonoid subgroup, named flavonols, of which quercetin and kaempferol are the most predominant components in plants [86]. Epicatechin, thought to be responsible for the main health effects of cocoa is another flavonoid compound found in unfermented cocoa beans. Epigallocatechin gallate (EGCG), formed by the ester of epigallocatechin and gallic acid, is present in green tea. Both of them have been associated with antioxidant and chemopreventive effects in several cell types [92,93]. Another flavonoid, narigenin, found in all citric fruits, seems to increase antioxidant defenses by limiting lipid peroxidation and protein carbonylation [85,94]. Lignans are non-flavonoid PE commonly found in grains, nuts, coffee and tea, cocoa, flaxseed, and some fruits [95]. According to some evidence, these PE are capable of mimicking the antioxidant effects of some hormones [96]. Finally, stilbenes are non-flavonoid PE of which the most studied is resveratrol, a compound with two phenolic rings connected by a styrene double bond, found in a wide variety of dietary foods, including grapes, wine, nuts, and berries [97,98,99]. Several in vitro and in vivo studies reported anti-cancer, antioxidant, anti-aging, anti-inflammatory and anti-pathogen properties of resveratrol [97,100,101].

Based on the results presented herein, these compounds may have some effects on the disease establishment. According to in vitro findings, 19 out of 22 studies reported the ability of PE to induce anti-proliferative, anti-inflammatory and proapoptotic effects on endometriotic cells. Only three studies did not find any positive effect exerted by PE in vitro [20,35,71]. Various mechanisms have been proposed to explain this in vitro action including the alteration of cell cycle proteins, the activation/inactivation of regulatory pathways, modification of ROS levels. Two considerations should be done in relation to the in vitro results obtained: 1. among the 22 published studies, nine were written by the same Chinese group [50,55,61,66,67,68,69,75,76]. Therefore, confirmatory findings by independent groups need to be obtained. 2. many studies have used cell lines as a model for endometriotic lesions. A number of immortalized cell lines deriving from endometriosis have been established by either forcing cells to survive through a cell crisis or by the introduction of one or more oncogene(s). However, genetic authentication and biological validation of these lines was disregarded by most authors. For instance, no STR profile was publicly available. Moreover, we have recently demonstrated that some of these endometriotic cell lines express ER-α but are PR-negative [8]. Since signaling initiated by both ER-α and PR is necessary for endometrial physiology, it is of foremost importance that cells are thoroughly characterized prior to each experiment for the maintenance of the proper phenotype and for their receptor status. This concept should be applied also to PE treatment of cells.

In line with in vitro findings, also results derived from animal models of endometriosis generally supported a beneficial effect of the compounds in reducing lesion growth and development. Indeed, a role of PE in limiting ectopic implants has been shown in 36 out of 38 studies independent of the specific drug used. Only two studies did not find any positive effect exerted by PE in in vivo experimental models [19,25] and both studies investigated the possible role of genistein in the treatment of induced models of endometriosis. Mechanisms proposed to explain this effect include decreased angiogenesis and microvessel density, enhanced fibrosis and apoptosis and alteration in MMP activity. Rats and mice offer attractive preclinical models of reproductive disorders because they are easily bred, they can be genetically manipulated, their reproductive system is well understood, and their small size means large quantities of drugs are not required for testing allowing for multiple replicates. In the context of endometriosis, these advantages apply but laboratory rats and mice do not exhibit spontaneous cyclical decidualization and menstruation. Therefore, although uterine tissue has been used to generate endometriosis-like lesions, the lesions are not formed from tissue undergoing active breakdown and remodeling as might be the case in women or menstruating primates. Therefore, similar to cell lines, experimental animal models of endometriosis are not devoid of limits. Due to their divergence from humans in key aspects of reproductive physiology, current experimental systems for the study of endometriosis are a very imperfect model [102]. As a matter of fact, most of the treatment for endometriosis used in experimental models provided satisfactory results while being of poor efficacy in humans [18,21,31,49,56,65,77].

As a matter of fact, the huge amount of in vitro and in vivo animal findings did not correspond to consistent literature in women affected. Randomized trials were only two using resveratrol and outcomes evaluated included pain score and MMP activity [49,65]. Quercitin was also shown to be able to reduce pain in a prospective cohort study [56]. Reasons for a limited reporting of PE effects in endometriosis patients is unclear. We cannot exclude that negative results have not been published. Alternatively, being natural compounds, they are viewed as dietary supplements and regularly prescribed with poor controls on outcomes. Certainly, based on results of experimental models, PE effect deserves to be investigated in more depth in future or ongoing clinical trials.

## 5. Conclusions

Phytoestrogens are naturally-occurring plant compounds that share a similar chemical structure and function to the estrogens found in the human body. Foods rich in phytoestrogens include soy, fruits, vegetables, spinach, sprouts, beans, cabbages, and grains. The effect of diet on hormonal activity, inflammatory markers, and the immune system means that the food choices women make might play a key role in the development of endometriosis. Furthermore, endometriosis has been shown to be related to prolonged exposure to the hormone estrogen in the absence of progesterone and to a prolonged inflammatory environment in the pelvis. Although there is consistent evidence, deriving from in vitro or in vivo animal model studies, for phytoestrogens’ biological properties in endometriosis, only a few studies have been published regarding their use in patients with endometriosis, with inconsistent results. Phytoestrogens have many favorable characteristics, such as anti-proliferative, anti-angiogenic, anti-inflammatory, pro-apoptotic and anti-oxidant properties, which could make them a viable alternative in the future for the control and prevention of endometriosis. More powered and well-designed trials are needed to better investigate PE effects in women affected by endometriosis.

## Figures and Tables

**Table 1:** Studies investigating phytoestrogen effect on endometriosis in experimental in vitro in human models.

Authors	Date	Substance	Cases	Controls	Results	Adverse Events
Edmunds et al. [20]	2005	Genistein, Daidzein, Naringenin or Chrysin (10^−4^–10^−9^ M)	EuSC from 11 women with endometriosis	EuSC from 7 women without endometriosis	- PE treatment did not attenuate aromatase activity in EuSC cultures from cases and controls - Genistein (10^−9^–10^−6^ M) increased aromatase activity in controls - Naringenin and Chrysin were potent inhibitors of aromatase in FCA - Genistein was inactive in FCA	Genistein consumption in reproductive age may have health risks
Wang et al. [28]	2011	Puerarin (10^−9^ M)	EcSC treated with Puerarin	EcSC treated with E2 (10^−8^ M) Untreated EcSC	- E2 showed a stimulatory effect on EcSC invasion compared with the untreated cells, but the combination of E2 with Puerarin reduced this effect - E2 treatment determined MMP-9 increase and TIMP-1 decrease, but the combination with Puerarin reversed this effect	NR
Cheng et al. [30]	2012	Puerarin (10^−9^ M)	EcSC treated with Puerarin +/− E2-BSA	EcSC treated with E2-BSA	- ERK1/2 (MAPK signaling) was highly activated by E2-BSA, which was reversed by Puerarin - E2-BSA induced the proliferation of EcSCs, which was reversed by Puerarin - Puerarin suppressed gene expression of Cyclin D1, COX-2 and cyp19	NR
Ji et al. [34]	2013	Puerarin (10^−9^ M)	EcSC treated with Puerarin +/− E2	EcSC treated with E2 +/− fulvestrant (anti-E2)	- Puerarin: suppressed proliferation of E2-stimulated EcSCs by increasing G1 phase of the cell cycle and down-regulating cyclin D1 and cdc25A expression changed recruitment pattern of nuclear receptor coregulators to estrogen receptor (less SRC-1 and SRC-3 coactivators but more NCoR and SMRT corepressors)	NR
Ricci et al. [35]	2013	Resveratrol (0, 25, 50 and 100 mM) EGCG (0, 20, 40, 80 and 100 mM).	EuEC from women with endometriosis	EuEC from women without endometriosis	- Both compounds induced reduction in EuEC proliferation and increased apoptosis in both groups - No significant difference in cell proliferation and apoptosis between cases and controls	NR
Matsuzaki et al. [40]	2014	EGCG (10^−9^ M)	EcSC and EuSC treated with ECGC (from 45 women with endometriosis)	EuSC and EuSC vehicle-treated or NAC(10 mM) treated (from 45 patients with endometriosis)	- EGCG: significantly inhibited cell proliferation, migration and invasion of both EuSC and EcSC significantly decreased the TGF-b1-dependent increase in the mRNA expression of fibrotic markers in both EuSC and EcSC Both EuSC and EcSC-mediated contraction of collagen gels were significantly attenuated at 8, 12 and 24 h after treatment with EGCG significantly inhibited TGF-ß1-stimulated activation of MAPK and Smad signaling pathways in both cells.	NR
Taguchi et al. [41]	2014	Resveratrol (10, 20 or 40 μM) (SIRT-1 activator) Sirtinol at 20 μM (SIRT-1 inhibitor)	EcSC	EuSC from patients without endometriosis	- No difference in the basal expression level of SIRT1 mRNA between EcSC and EuSC - Resveratrol: suppressed TNF-α-induced IL-8 release from EcSC in a dose-dependent manner while Sirtinol increased IL-8 release had increased anti-inflammatory effects on EcSC than on EuSC	NR
Taguchi et al. [47]	2016	Resveratrol (40–120 mΜ) TRAIL 100 ng/mL	EcSC treated with resveratrol and TRAIL	EcSC treated with TRAIL	- Resveratrol: alone did not induce apoptosis in EcSC, but significantly reduced survivin mRNA expression enhanced TRAIL-induced apoptosis	NR
Kim et al. [48]	2017	PFE (25, 50, and 100 μg/mL) containing Genistein, Daidzein, Kakkalide, Puerarin, Tectoridin	Human endometriotic (11Z and 12Z) and mesothelial (Met5A) cells treated with PFE	Human endometriotic (11Z and 12Z) and mesothelial (Met5A) cells not treated with PFE	- PFE: inhibited endometriotic cell adhesion to mesothelial cells inhibited endometriotic cell migration at 100 μg/mL inhibited RNA and protein expression of MMP-2 and MMP-9 and increased the phosphorylation of ERK1/2 in endometriotic cells	NR
Park et al. [50]	2017	Narigenin (100 μM)	VK2/E6E7 and End1/E6E7 cells treated with Narigenin	VK2/E6E7 and End1/E6E7 cells not treated with Narigenin	- Narigenin: decreased proliferation and increased apoptosis increased ROS production increased apoptosis through generation of ER stress regulatory genes, activation of MAPK signaling and inactivation of PI3K pathway	NR
Park et al. [55]	2018	Apigenin (20 μM)	VK2/E6E7 and End1/E6E7 cells treated with Apigenin	VK2/E6E7 and End1/E6E7 cells not treated with Apigenin	- Apigenin: decreased cell proliferation, reduced MMP expression increased apoptosis inducing mitochondrial pro-apoptotic proteins, Bax, Bak and Cyt c in End1/E6E7 cells increased concentrations of calcium ions in the cytosol and ROS generation with lipid peroxidation induced ER stress by increasing phosphorylation of unfolded proteins inhibited the phosphorylation of ERK1/2 in both cell lines, but the phosphorylation of AKT increased only in End1/E6E7 cells	NR
Takaoka et al. [57]	2018	DRIAs (0.2, 2, 20 μM)	EcSC from 24 patients with endometriosis	EuSC from 12 patients without endometriosis	- DRIAs: inhibited proliferation of EcSCs in a concentration-dependent manner, but not of EuSCs decreased IL-6, IL-8, COX-2 and aromatase mRNA levels, PGE2 protein levels and aromatase enzyme activity suppressed TNF-α-induced IκB expression, the NF-kB-ikb complex formation, and the uptake of p65 into the nucleus	NR
Arablou et al. [59]	2019	Resveratrol (100 μM)	13 EuSC 8 EcSC from 40 women with endometriosis	11 EuSC from 15 women without endometriosis	- Basal expression of IGF-1 and HGF gene were significantly higher in EcSC - Resveratrol: decreased IGF-1 and HGF gene expression more in EuSC from control women than in EcSC and EuSC from women without endometriosis decreased IGF-1 and HGF protein production in EuSC from women with endometriosis and EcSC	Resveratrol at 200- and 400-μM concentrations
Ham et al. [61]	2019	Silibinin (0,2, 5, 10, 25,50 μM)	VK2/E6E7 and End1/E6E7 cells treated with Silibinin	EuSC treated with Silibinin	- Silibinin: decreased proliferation of endometriotic cells but not of EuSC induced cell cycle arrest and apoptosis in endometriotic cells with an increase of the sub-G1 population in a dose-dependent manner increased ROS levels and lipid peroxidation in endometriotic cells stimulated ER stress through the disruption of calcium homeostasis in cytosol and mitochondrial matrix in endometriotic cells causing cell death induced changes in the MAPK signaling pathway in VK2-End1cells	NR
Ryu et al. [66]	2019	Chyrisin (0,5,10,20,50, 100 μM	VK2/E6E7 and End1/E6E7 cells treated with Chyrisin	VK2/E6E7 and End1/E6E7 cells not treated with Chyrisin EuC	- Chyrisin: decreased proliferation and stimulated apoptosis and cell cycle arrest in the sub-G1 phase increased cytosolic calcium levels and ROS production activated ER stress by stimulating the unfolded protein response proteins inactivated PI3K/PKB signaling pathway in a dose-dependent manner	NR
Park et al. [67]	2019	Delphinidin (0,5,10,20,50,100 μM)	VK2/E6E7 and End1/E6E7 cells treated with Delphinidin	VK2/E6E7 and End1/E6E7 cells not treated with Delphinidin	- Delphinidin: decreased cell proliferation at 100 μM. In both cell lines, the percentage of cells in the sub G0/G1 stage gradually increased with increase of Delphinidin concentration increased levels of cytosolic calcium ions and mitochondrial depolarization inactivated PI3K/AKT and ERK1/2 and increased the phosphorylation of P38 MAPK and P90RSK proteins in both cell lines- Cells in late apoptosis increased by 1016%	NR
Park et al. [68]	2019	Quercetin (0, 2, 5, 10, 20,50 μM)	VK2/E6E7 and End1/E6E7 cells treated with Quercetin	VK2/E6E7 and End1/E6E7 cells not treated with Quercetin	- Quercetin: decreased cell proliferation at 20 μM. The percentage of G0/G1 stage cells increased in VK2/E6E7 cells although there was a decrease in End1/E6E7 cells increased ROS production in both cell types decreased phosphorylation of ERK1/2, P90RSK, P38, AKT, P70S6K, and S6 proteins - Cells in late apoptosis increased for both cell lines - The loss of MMP increased to 2300% in VK2/E6E7 cells and 670% in End1/E6E7 cells at 20 μM	NR
Park et al. [69]	2019	Luteolin (0, 5, 10, 20, 50 and 100 μM)	VK2/E6E7 and End1/E6E7 cells treated with luteolin	VK2/E6E7 and End1/E6E7 cells not treated with luteolin	- Luteolin: decreased proliferation in a dose-dependent manner. induced cell cycle arrest in the sub-G0/G1 stage and decreased the cell percentage in the G0/G1 stage in both cell lines increased cell apoptosis through cytosolic calcium regulation and ROS accumulation inhibited the phosphorylation of ERK1/2, JNK and PI3K/AKT signal proteins while activating P38 MAPK proteins	NR
Hernandes et al. [71]	2020	Rutin and extract of Uncaria guianensis	EuSC and EcSC from 4 women with endometriosis	EuSC from 2 women without endometriosis	- Increased ROS levels in EuC from controls treated with ALE, ABE, and ARE and in EuC of patients with endometriosis treated with Rutin, ARE, Rutin + ALE, and Rutin + ARE - Increased ROS levels in EcC treated with ALE - Increased IL-15, IL-17A, IL-4, IL-6, TNF-alfa and VEGF levels in EuC from controls treated with ABE - Increased EGF in EcC treated with ALE	
Khazaei et al. [74]	2020	Resveratrol (0, 10, 50, 100, 200 μM)	EcSC from 9 patients with endometriosis	EuSC from 9 patients without endometriosis	- Resveratrol (200 μM) completely inhibited growth and angiogenesis in both cells types in a dose-dependent manner - NO level was higher in endometriotic cells. Resveratrol reduced NO level in both endometriotic and endometrial cells - Effect on apoptotic genes (P53, Bax, Bcl2 and caspase 3) and SIRT1	NR
Park et al. [75]	2020	DMF (Chyrisin) (0, 20, 50, 100 μM)	VK2/E6E7 and End1/E6E7 cells treated with DMF	VK2/E6E7 and End1/E6E7 cells not treated with DMF	- DMF: induced sub-G1 cell cycle arrest in VK2/E6E7 cell, while End1/E6E7 cells were arrested at the G2/M phase decreased proliferation and induced apoptosis in both cell lines disrupted mitochondrial regulation and increased ROS production and lipid peroxidation in both cell lines, increasing ER stress-response pathways down-regulated ERK1/2 pathway in End1/E6E7 cells but upregulated it in VK2/E6E7 cells inhibited PI3K/AKT pathway in both cell lines	
Park et al. [76]	2020	Myricetin (0, 5, 10, 20, 50, 100 μM)	VK2/E6E7 and End1/E6E7 cells treated with Myricetin	VK2/E6E7 and End1/E6E7 cells not treated with Myricetin	- Myricetin decreased proliferation in a dose-dependent manner, caused by cycle arrest at the sub G0/G1 phase and increased late apoptosis induced depolarization of the mitochondrial membrane, increased level of cytosolic calcium ions in the cells and ROS generation and accumulation in the cytoplasm down-regulated the phosphorylated ERK1/2 and PI3K/AKT signal proteins, induced p38 protein activation in a dose-dependent manner	NR

Only *p*-values statistically significant (*p* < 0.05) were reported. *Legend:* EuSC = eutopic endometrial stromal cells; PE = phytoestrogen; FCA = free cell assay; EcSC = ectopic endometrial stromal cells; E2 = 17 beta-estradiol; MMP = matrix metalloproteinase; TIMP = tissue inhibitors of metalloproteinases; NR = not reported; BSA = bovine serum albumin; ERK = extracellular signal-regulated kinases; MAPK = mitogen-activated protein kinase; COX-2 = cyclooxygenase-2; cyp19 = cytochrome P450 19; EGCG = epigallocatechin gallate; EuEC = eutopic endometrial epithelial cells; cdc25a = cell division cycle 25 homolog A; SRC = steroid receptor coactivator; NCoR = nuclear receptor corepressor; SMRT = silencing mediator for retinoid or thyroid-hormone receptors; SIRT-1 = sirtuin 1; TNF alfa = tumor necrosis factor alfa; IL = interleukin; TRAIL = TNF alfa related-apoptosis-inducing ligand; PFE = Pueraria flowers extract; VK2 = vaginal mucosa-derived epithelial endometriotic cell; End1/E6E7 = endocervix epithelial-derived endometriotic cell line; ROS = reactive oxygen species; ER = endoplasmic reticulum; GADD153 = G1 arrest and DNA damage 153; IRE1α: inositol-requiring protein 1α; GRP78: the 78-kDa glucose-regulated protein; PI3K = phosphatidylinositol 3-kinase; DRIA: daidzein-rich isoflavone aglycones; Bax = Bcl-2-associated X protein; Bak = Bcl-2-antagonist/killer; Cyt C = cytochrome complex; PERK = PRKR-like ER kinase; eIF2α: eukaryotic translation initiation factor 2α; PG = prostaglandin; NF-kB-ikb = nuclear factor-κB-IKB-inhibitory proteins; EuC = eutopic endometrial cells; IGF-1: insulin-like growth factor-1; HGF: hepatocyte growth factor; NO = nitric oxide; Bcl 2 = B-cell lymphoma 2; DMF: 5,7-dimethoxyflavone; EcC = ectopic endometrial cells; ABE = aqueous bark extract of U. guianensis; ALE = aqueous leaf extract of U. guianensis; ARE = aqueous root extract of U. guianensis; EGF = epidermal growth factor; NAC = N-acetyl-L-cysteine; TGF-b1 = tumor growth factor-b1; Smad protein = small mother against decapentaplegic protein.

**Table 2:** Studies investigating effects of non-oral intake of phytoestrogens on endometriosis in an animal in vivo model.

Authors	Date	Model	Substance	Cases (n)	Control (n)	Results
Cotroneo et al. [19]	2001	Rats	S.C, genistein: 50 μg/g(high) 16.6 μg/g (average) 5 μg/g(low)	7/8/10	Vehicle (20) or Estrone (7)	Higher and average dose of Genistein and administration of estrone: - increased uterine/body weight ratios - increased uterine PR expression at all doses - supported growth of the implanted tissue in a dose-responsive manner
Laschke et al. [23]	2008	Hamsters	I.P. EGCG 65 mg/kg	7	Vehicle (10)	- inhibited angiogenesis and blood perfusion of endometriotic lesions
Xu et al. [24]	2009	Mice	I.P. EGCG 50 mg/kg	10	Vitamin E (10) Vehicle (10)	- smaller lesions than control animals - down-regulation of VEGFA mRNA expression - down-regulation of MAPK1 and NFKB mRNA expression
Laschke et al. [25]	2010	Hamsters	I.P genistein 50/200 mg/kg	Low dose (6) High dose (4)	Vehicle (6)	- blood perfusion and angiogenesis of endometriotic lesions was not affected by Genistein treatment
Xu et al. [29]	2011	Mice	I.P. EGCG 50 mg/kg	10	Vitamin E (10) Vehicle (10)	- decreased lesion size - down-regulation of MMP-9, CXCL3, VEGFC, c-JUN, and IFNγ - suppression of VEGFC mRNA and protein - decreased VEGFC levels in both microvessels and glandular epithelial cells
Ergenoglu et al. [33]	2013	Rats	I.M. resveratrol 10 mg/kg	6	Vehicle (6)	- reduction of implant size - decreased levels of VEGF in peritoneal fluid and plasma - decreased levels of MCP-1 in peritoneal fluid - suppression of VEGF expression in endometriotic tissue
Ricci et al. [35]	2013	Mice	I.P. resveratrol 10–25 mg/kg; EGCG 20–100 mg/kg by esophageal gavage	Resveratrol (29) EGCG (27)	Vehicle (NR)	- both treatments reduced number and volume of lesions - both diminished proliferation and vascular density of endometriotic lesions - both increased apoptosis
Wang et al. [37]	2013	Mice	I.P. EGCG 50 mg/kg or pro-EGCG 50 mg/kg	EGCG (8) proEGCG (8)	Vitamin E (8) Vehicle (8)	- decreased lesion size - decreased angiogenesis - increased total apoptopic cell numbers
Amaya et al. [38]	2014	Mice	S.C. resveratrol 6/30/60 mg/kg	E2 + 6 mg of Resveratrol (4) E2 + 30 mg of Resveratrol (4) E2 + 60 mg of Resveratrol (4)	E2 (4) E2 + P (4)	- reduction in ESR1 and Ki-67 by the highest dose in eutopic endometrial epithelial cells - reduction in Ki-67 expression by the highest dose in endometrial stroma
Matsuzaki et al. [40]	2014	Mice	I.P. EGCG 50 mg/kg	NR	NR	- lower scores for both Sirius red and Masson trichrome staining
Yavuz et al. [42]	2014	Rats	I.P resveratrol 1/10 mg/kg	Low dose (8) High dose (8)	Vehicle (8)	- lower implants volume in cases independently from dose - reduced oxidative stress in cases compared to controls in a dose-dependent manner - proliferative scores for glandular tissue and stromal tissue were lower in cases
Bayoglu Tekin et al. [43]	2015	Mice	I.M. resveratrol 30 mg/kg S.C. 1 mg/kg single dose LA	Resveratrol (NR) LA and resveratrol (NR)	Vehicle (NR) LA (NR)	- reduced implant volumes, histopathological grade and immuno-reactivity to MMP-2, MMP-9 and VEGF - decreased plasma and peritoneal fluid levels of IL-6, IL-8 and TNF-α
Singh et al. [45]	2015	Mice	I.P. EGCG and doxycycline (NPs) at a dose of 40 mg/kg body weight	50	10	- decreased ROS and LPO, MMP-2 and MMP-9 activity - decreased angiogenesis and microvessel density
Jouhari et al. [52]	2018	Rats	S.C. 100 mg/kg silymarin	8	Vehicle (8) Letrozole (8) Cabergoline (8)	- smaller volume of implants - lower mean score of the histopathological evaluation of the implants
Wei et al. [58]	2018	Mice	I.P. nobiletin 10, 20 mg/kg	Low dose Nobiletin (3) High dose Nobiletin (3)	3 (endometriosis) 3 (sham)	- reduced lesion size - lower PCNA and VEGF immunostaining - higher E-cadherin staining - decreased levels of IL-6, IL-1β, and MMP-3 - reduced levels of TNF-α and MMP-1 - reduced phosphorylation of IKKα, IκBα and p65 factors
Ding et al. [60]	2019	Mice	I.P. scutellarin 15 mg/kg 7.5 mg/kg	Low dose (9) High dose (9)	Vehicle (9)	- reduction of lesion weight, improved hyperalgesia, reduced proliferation, angiogenesis, and fibrogenesis of the lesions - reduced the platelet activation rate in peripheral blood
Ham et al. [61]	2019	Mice	I.P. silibinin 100 μL	15	Vehicle (15)	- reduced average size of lesions - decreased expression of TNF-α, IL-1β, and IL-6 mRNA
Park et al. [68]	2019	Mice	I.P. quercetin 35 mg/kg	15	Vehicle (15	- decreased lesion volume - decreased Ccnd1 mRNA
Park et al. [69]	2019	Mice	I.P. luteolin 40 mg/kg/day	6	Vehicle (6)	- reduced endometriotic lesions growth - decreased mRNA expression of Ccne1, Cdk2 and Cdk4
Park et al. [76]	2020	Mice	I.P. myricetin 30 mg/kg	10	Vehicle (10)	- decreased lesion size - decreased Ccne1 mRNA expression

Only statistically significant effects (*p* < 0.05) were reported. *Legend*: S.C = subcutaneous; PR = progesterone receptor; I.P = intraperitoneal; EGCG = epigallocatechin-3-gallate; E2 = 17b-estradiol; NR = not reported; VEGF = vascular endothelial growth factor; MAPK1 = mitogen activated protein kinase 1; NFKB = nuclear factor kappa B; MMP = matrix metalloproteinase; CXCL3 = chemokine (C-X-C motif) ligand 3; IFNγ = interferon γ; I.M. = intramuscular; MCP-1 = monocyte chemotactic protein 1; proEGCG = prodrug of green tea epigallocatechin-3-gallate; P = progesterone; ESR1 = estrogen receptor α; LA = leuprolide acetate; IL = interleukin; TNF-α = tumor necrosis factor-α; NPs = synthesized nanoparticles; ROS = reactive oxygen species; LPO = lipid peroxidation; EM = endometriosis; PCNA = proliferating cell nuclear antigen; IKKα = IκB kinase; Ccnd1 = cyclin D1; DMSO = dimethyl sulfoxide; Ccne1 = cyclin e1; Cdk = cyclin-dependent kinase.

**Table 3:** Studies investigating the effects of phytoestrogen oral intake on endometriosis in an animal in vivo model.

Authors	Date	Model	Substance	Cases (n)	Control (n)	Results
Cotroneo et al. [19]	2001	rats	Genistein 250/1000 mg/kg AIN-76A diet	12 + 11 (lower/higher dietary intake)	Vehicle (17)	- increased uterine PRB by the higher dietary intake
Yavuz et al. [22]	2007	rats	Genistein 500 mg/kg	10	Raloxifene at 10 mg/kg or no vehicle (10 + 13)	- smaller area and lower histological scores of endometriotic lesions
Bruner-Tran et al. [26]	2011	mice	Resveratrol 6 mg	20	Vehicle (16)	- decreased number of endometrial implants per mouse by 60% and the total volume of lesions per mouse by 80%
Chen et al. [27]	2011	rats	Puerarin High (600 mg/kg) Medium (200 mg/kg) Low (60 mg/kg)	45 (15 each)	Danazol at dose of 80 mg/kg or vehicle (15 + 15)	- inhibition of growth of ectopic implants for both Puerarin and Danazol - inhibition of P450 aromatase expression and reduction of estrogen levels in endometriotic tissue using the low dose
Rudzitis-Auth et al. [32]	2012	mice	Xanthohumol 100 mM	8	Vehicle (8)	- decreased lesion growth and volume - suppression of peritoneal and mesenteric endometriotic lesions - reduced proliferation and PI3-J protein
Rudzitis-Auth et al. [36]	2013	mice	Resveratrol 40 mg/kg	10	Vehicle (10)	- lower lesion volume and size - reduced number of PCNA-positive stromal cells - lower Ki-67-positive glandular cells - inhibition of angiogenesis
Ricci et al. [35]	2013	mice	EGCG 20 or 100 mg/kg	18 (9 each)	Vehicle (9)	- reduction of mean number and volume of endometriotic lesions - reduced vascular density - increased apoptosis
Demirel et al. [39]	2014	rats	Extract of *Achillea bierbersteinii* N-Hexane EtOAc MeOH	18	Vehicle or 6 buserelin acetate 20 mg/weekly sc (12)	- decreased endometriotic volume in EtOAc and buserelin groups - reduced peritoneal TNF-α in EtOAc and reference, VEGF in both and IL-6 in EtOAc
Ozcan Censoy et al. [44]	2015	rats	Resveratrol 60 mg/kg/day	7	Vehicle or leuprolide acetate at 1 mg/kg depot (7 + 8)	- Both resveratrol and leuprolide acetate - reduced mean surface areas of endometriotic implants - reduced VEGF score in endometriotic implants - reduced peritoneal and serum VEGF and MCP-1
Di Paola et al. [46]	2016	rats	mPEA\PLD 10 mg/kg	5	Vehicle (5)	- decreased cyst diameter, histological injury score, mast cells number and VEGF, ICAM-1 expression - increased fibrosis score and NGF - less pain behaviors
Ferella et al. [51]	2018	mice	Wogonin 20 mg/kg/day	12	Vehicle (11)	- increased percentage of apoptotic cells
Nahari et al. [54]	2018	rats	Sylimarin (SMN) 50 mg/kg/day	6	Vehicle (6)	- decreased endometriotic-like lesions size and percentage of cell proliferation, angiogenesis, GDNF, grfα and Bcl-6b. - enhanced fibrosis and apoptosis - enhanced ERK1/2 expression.
Melekoglu et al. [53]	2018	rats	Nerolidol 100 mg/kg or Hesperidin 50 mg/kg	16 (8 each)	(8)	- lower volume, more evident in nerolidol group - increased GSH, SOD and GPx
Takaoka et al. [57]	2018	mice	DRIA food at 0.06%	NR	Vehicle (NR)	- decreased number, weight and Ki-67 proliferative activity of endometriotic-like lesions - decreased IL-6, IL-8 and COX-2
Ilhan et al. [62]	2019	rats	Extract of *Urtica dioica* N-Hexane EtOAc MeOH - Fraction A - Fraction B - Fraction C - Fraction D	18 (6 each)	Vehicle or buserelin acetate 20 mg/weekly sc (12)	- decreased adhesion score, endometriotic implant volume, peritoneal TNF-α, VEGF and IL-6 in MeOH, reference and Fraction C
Ilhan et al. [63]	2019	rats	Extract of *Anthemis austriaca* N-Hexane EtOAc MeOH - Fraction A - Fraction B - Fraction C (Quercetin and Apigenin) - Fraction D	18 (6 each)	Vehicle or buserelin acetate 20 mg/weekly sc (12)	- decreased endometriotic implant volume and adhesion score, peritoneal TNF-α, VEGF and IL-6 in EtOAc, MeOH, and reference - decreased adhesion score and endometriotic implants volume and peritoneal IL-6 and VEGF in Fraction A and in Fraction C - decreased peritoneal TNF-α in Fraction C
Kapoor et al. [64]	2019	rats	Narigenin 50 mg/kg/day: Only the day of endometriosis induction Every day for 21 days	12 (6 each)	oral dienogest at dose of 0.3 mg/kg/day for 21 days or nothing (12 endometriosis)(6 sham controls)	Both Narigenin and Dienogest: - suppression of endometriotic lesion growth and reduced lesion weight by inducing apoptosis, cellular ROS and damaging mitochondrial membrane. - inhibition of NO release and restoration of TNFα level - reduced TAK1 levels by 3-fold at dose of 1 µM and 5 µM, reduced VEGF by 2 and 4-fold at dose of 1 µM and 5 µM - mitigation of the expression of Nrf2, its repressor and effector molecule reduced number of cells migrating at 1 µM and 5 µM - reduced expression of MMP-2 and MMP-3
Bina et al. [70]	2020	rats	Achillea cretica (A.C.) extract once a day at dose of 100 mg/kg/day; 200 mg/kg/day; 400 mg/kg/day	18 (6 each)	Vehicle or letrozole (12 endometriosis) (6 sham controls)	- reduced size of implanted tissue, mean score of the histopathological evaluation of the implants, thickness of epithelial layer. - decreased serum TNF-α and both serum and tissue IL-6 levels after treatment with A.C. 100, 400 and letrozole. - reduced tissue TNF-α and both serum and tissue VEGF levels after treatment with A.C. 100 and letrozole
Hsu et al. [72]	2020	mice	ISL and estrogens (10 mg/kg/day) 1 mg/kg (LI) 5 mg/kg (HI)	12 (6 each)	Vehicle (6)	- smaller volume of lesions - decreased tissue VEGF level in HI - decreased serum IL-1β in HI cand decreased tissue IL-1β in HI e LI. - decreased serum and tissue IL-6 levelincreased Bax, cleaved-caspase-3 and E-Cadherin expression in HI. - decreased bcl-2, ER-β, N-Cadherin, Snail and Slug expression.
Ilhan et al. [73]	2020	rats	Extract of *Melilotus officinalis* (kaempferol, quercetin, and coumarin derivatives) at 100 mg/kg/day N-Hexane EtOAc MeOH - Fraction A - Fraction B - Fraction C - Fraction D	18 (6 each)	Vehicle or buserelin acetate 20 mg/weekly sc (12)	Both MeOH, Fraction C and buserelin acetate: - decreased endometriotic implants volume and peritoneal TNF-α, VEGF and IL-6 levels - decreased endometriotic implant adhesion score, volume and IL-6 in Fraction B

Only statistically significant effects (*p* < 0.05) were reported. *Legend*: PRB, progesterone receptor type B; EcSC = ectopic endometrial stromal cells; EGCG = epigallocatechin gallate; EtOAc = ethyl acetate; MeOH = methanol; TNF alfa = tumor necrosis factor alfa; VEGF = vascular endothelial growth factor; IL = interleukin; MCP-1 = monocyte chemoattractant protein-1; mPEA\PLD = micronized palmitoylethanolamide/polydatin; ICAM-1 = intercellular adhesion molecule-1; NGF = nerve growth factor; GDNF = glial cell line-derived neurotrophic factor; grfα = receptor of GDNF; ERK1/2 = extracellular signal-regulated kinases; GSH = glutathione; SOD = superoxide dismutase; GPx = glutathione peroxidase; DRIA = daidzein-rich isoflavone aglycones; NR = not reported; COX = cyclooxygenase; ROS = reactive oxygen species; NO = nitric oxide; Nrf2 = nuclear factor erythroid 2–related factor; MMP = matrix metalloproteinases; ISL = isoliquiritigenin; HI = high dose of ISL; LI = low dose of ISL; ER = estrogen receptor; H&E = hematoxylin and eosin; PCNA = proliferating cell nuclear antigen.

**Table 4:** Studies investigating the effect of phytoestrogen oral intake on humans.

Authors	Date	Study Design	Substance and Duration	Age (Years, Mean)	Case (n)	Control (n)	Results
Nagata et al. [18]	2001	prospective cohort study	Genistein, Daidzein in one year	35–54 42.9 ± 4.4	1172	n.a.	- decreased risk of hysterectomy for pain: RR (95% CI) 0.35 (0.13 ± 0.97)
Tsuchiya et al. [21]	2007	case-control study	Urinary levels of Genistein/Daidzein, NR	20–45 Stage I–II: 32.3 ± 3.2 Stage III–IV: 32.6 ± 3.7	79 (stage I–II *n* = 31; stage III–IV *n* = 48)	59	- inversely associated with stage III-IV with aOR 0.21 (95% CI = 0.06–0.76) for Genistein and 0.29 (0.08–1.03) for Daidzein levels - ER-2 RsaI R/r + R/R genotype more frequent than the r/r genotype in advanced stages
Maia et al. [31]	2012	retrospective study	Resveratrol 30 mg for 2–6 months	24–40 31 ± 4	OC+ resveratrol (26)	OC (16)	- reduction in pain scores, with 82% of patients reporting complete resolution of dysmenorrhea and pelvic pain after 2 months - lower COX-2 expression in eutopic endometrium at immunohistochemistry - lower aromatase expression in eutopic endometrium at immunohistochemistry
Mendes da Silva et al. [49]	2017	randomized clinical trial	Resveratrol 40 mg for 42 days	20–50 35.4 ± 7.1	22	Placebo (22)	- no difference in pain scores between groups [median difference: 0.75, 95% confidence interval: −1.6 to 2.3]
Signorile et al. [56]	2018	prospective cohort study	Quercetin 200 mg, titrated Turmeric 20 mg, titrated Parthenium 19.5 mg for three months	34 ± NR	Group I (30 patients treated with all the ingredients); Group II (30 patients treated with only linseed oil and 5 MTHF calcium salt)	Group III, placebo (30)	- significant reduction of headache (from 14% to 4%), cystitis (from 12% to 2%), muscles ache (from 4% to 1%), irritable colon (from 15% to 6%), dysmenorrhea (from 62% to 18%) and dyspareunia (from 30% to 15%), CPP (from 62% to 18%) - reduction of serum PGE2 level
Kodarahmian et al. [65]	2019	placebo-controlled, parallel, randomized double-blind exploratory clinical trial	ResveratroL 400 mg for 12–14 weeks	18–37 30.19 ± 2.4	17	Placebo (17)	- reduced MMP-2 and MMP-9 mRNA and protein levels in eutopic endometrium - reduced level of MMP-2 and MMP-9 in endometrial fluid and serum
Youseflu et al. [77]	2020	case-control study on dietary data	Isoflavones, lignans, coumestrol, in one year	15–45 yo 31.01 ± 6.56	78	78	- reduced risk of endometriosis for Isoflavones [OR 0.38 (0.33–0.83)], Lignan [OR 0.49 (0.46–0.52)], and Coumestrol [OR 0.38 (0.15–0.96)] assumption

Only statistically significant effects (*p* < 0.05) were reported. *Legend*: n.a = not applicable; RR = rate ratios; CI = confidence interval; aOR = adjusted odds ratio; ER-2 = estrogen receptor-2; LPS = laparoscopy; HYS = hysteroscopy; OC = oral contraceptive; COX-2 = cyclo-oxygenase-2: SD = standard deviation; MTHF = methyltetrahydrofolate; CPP = chronic pelvic pain; PGE2 = prostaglandin E2; MMP = matrix metalloproteinase, NR = not reported. According to Maia and coworkers, the addition of 30 mg of resveratrol to the oral contraceptives (OC) regimen resulted in a further significant reduction in pain scores after 2 months of treatment, with complete resolution of dysmenorrhea and pelvic pain reported in 82% of cases [31]. Additionally, COX-2 and aromatase expression were significantly lower in the eutopic endometrium of patients using the combination of OC with resveratrol compared with those using OC alone [31]. Kodarahmian and colleagues investigated the effects of resveratrol on MMP-2 and MMP-9 levels in endometriosis patients (*n* = 34) who were randomly divided into treatment *(n* = 17 patients treated with 400 mg of resveratrol) and control (*n* = 17 patients treated with placebo) women. Reduced levels of both MMP-2 and MMP-9 mRNA and protein were found in eutopic endometrium as well as lower concentrations in serum and endometrial fluid following the administration of resveratrol for 12–14 weeks [65]. A randomized controlled trial conducted by da Mendes da Silva and colleagues randomized subjects to receive monophasic OC for 42 days in addition to 40 mg of resveratrol or placebo in order to compare them for the reduction of pain scores. In contrast with other studies, pain scores after treatment were not significantly different between groups leading the authors to conclude that daily use of resveratrol combined with continuous use of a OC, was not superior to a OC alone for the treatment of pain in women with endometriosis [49]. The study conducted by Signorile et al., evaluated the effects of quercetin, titrated turmeric and titrated parthenium in a dietary supplement with linoleic acid, alpha linolenic acid, nicotinamide and 5-methyltetrahydrofolate calcium salt in patients affected by endometriosis. The authors found a significant reduction of headache, cystitis, muscles ache, irritable colon, dysmenorrhea, dyspareunia and chronic pelvic pain (CPP) in treated patients compared to patients treated with a composition comprising only of linseed oil and 5-methyltetrahydrofolate calcium salt and to the placebo group. Moreover, they reported reduction of serum dosage of PGE2 in patients treated with the dietary supplements for three months [56]. A case control study collected dietary data from 78 women with a laparoscopically confirmed endometriosis and 78 patients with normal pelvis using a food frequency questionnaire (FFQ) as a validated semi-quantitative questionnaire and analyzing PE type in each dietary item. The logistic regression model observed inverse associations between the consumption of PE, total isoflavones (especially related to formononetin and glycitein) and endometriosis risk. Additionally, high consumption of lignans (secoisolariciresinol, lariciresinol, matairesinol) and coumestrol in the third quartile resulted in a reduced risk of endometriosis. The authors concluded supporting the role of PE consumption in limiting the progression of endometriosis due to its inflammatory nature and the hormonal basis of the disease [77].

## Data Availability

Data sharing is not applicable to this article as no new data were created or analyzed in this study.

## Supplementary Materials

The following are available online at https://www.mdpi.com/article/10.3390/nu13082532/s1, Figure S1: Risk of bias assessment according to the risk of bias tool by Clarity Group [79].

## References

[1] Parazzini F., Esposito G., Tozzi L., Noli S., Bianchi S. (2017). Epidemiology of endometriosis and its comorbidities. Eur. J. Obstet. Gynecol. Reprod. Biol..

[2] Vercellini P.P., Vigano P., Somigliana E., Fedele L. (2013). Endometriosis: Pathogenesis and treatment. Nat. Rev. Endocrinol..

[3] Vigano P., Candiani M., Monno A., Giacomini E., Vercellini P.P., Somigliana E. (2018). Time to redefine endometriosis including its pro-fibrotic nature. Hum. Reprod..

[4] Vigano P., Ottolina J., Bartiromo L., Bonavina G., Schimberni M., Villanacci R., Candiani M. (2020). Cellular components contributing to fibrosis in endometriosis: A literature review. J. Minim. Invasive Gynecol..

[5] Huhtinen K., Stahle M., Perheentupa A., Poutanen M. (2012). Estrogen biosynthesis and signaling in endometriosis. Mol. Cell Endocrinol..

[6] Kyama C.M., Debrock S., Mwenda J.M., D’Hooghe T.M. (2003). Potential involvement of the immune system in the development of endometriosis. Reprod. Biol. Endocrinol..

[7] Zondervan K.T., Becker C.M., Koga K., Missmer S.A., Taylor R.N., Viganò P. (2018). Endometriosis. Nat Rev Dis Primers.

[8] Romano A., Xanthoulea S., Giacomini E., Delvoux B., Alleva E., Vigano P. (2020). Endometriotic cell culture contamination and authenticity: A source of bias in in vitro research?. Hum. Reprod..

[9] Stratton P., Berkley K.J. (2011). Chronic pelvic pain and endometriosis: Translational evidence of the relationship and implications. Hum. Reprod. Update.

[10] Goncalves G.A. (2018). p27(kip1) as a key regulator of endometriosis. Eur. J. Obstet. Gynecol. Reprod. Biol..

[11] Harris H.R., Chavarro J.E., Malspeis S., Willett W.C., Missmer S.A. (2013). Dairy-food, calcium, magnesium and vitamin D intake and endometriosis: A prospective cohort study. Am. J. Epidemiol..

[12] Harris H.R., Eke A.C., Chavarro J.E., Missmer S.A. (2018). Fruit and vegetable consumption and risk of endometriosis. Hum. Reprod..

[13] Parazzini F., Viganò P., Candiani M., Fedele L. (2013). Diet and endometriosis risk: A literature review. Reprod. Biomed. Online.

[14] Nikolić I.L., Savić-Gajić I.M., Tačić A.D., Savić I.M. (2017). Classification and biological activity of phytoestrogens: A review. Adv. Technol..

[15] Roca P., Sastre-Serra J., Nadal-Serrano M., Pons D.G., Blanquer-Rossello M.D., Oliver J. (2014). Phytoestrogens and mitochondrial biogenesis in breast cancer. Influence of estrogen receptors ratio. Curr. Pharm. Des..

[16] Kirichenko T.V., Myasoedova V.A., Orekhova V.A., Ravani A.L., Nikitina N.A., Grechko A.V., Sobenin I.A., Orekhov A.N. (2017). Phytoestrogen-rich natural preparation for treatment of climacteric syndrome and atherosclerosis prevention in Perimenopausal women. Phytother. Res..

[17] Shukla V., Chandra V., Sankhwar P., Popli P., Kaushal J.B., Sirohi V.K., Dwivedi A. (2015). Phytoestrogen genistein inhibits EGFR/PI3K/NF-kB activation and induces apoptosis in human endometrial hyperplasial cells. RSC Adv..

[18] Nagata C., Takatsuka N., Kawakami N., Shimizu H. (2001). Soy product intake and premenopausal hysterectomy in a follow-up study of Japanese women. Eur. J. Clin. Nutr..

[19] Cotroneo M.S., Lamartiniere C.A. (2001). Pharmacologic, but not dietary, genistein supports endometriosis in a rat model. Toxicol. Sci..

[20] Edmunds K.M., Holloway A.C., Crankshaw D.J., Agarwal S.K., Foster W.G. (2005). The effects of dietary phytoestrogens on aromatase activity in human endometrial stromal cells. Reprod. Nutr. Dev..

[21] Tsuchiya M., Miura T., Hanaoka T., Iwasaki M., Sasaki H., Tanaka T., Nakao H., Katoh T., Ikenoue T., Kabuto M. (2007). Effect of soy isoflavones on endometriosis: Interaction with estrogen receptor 2 gene polymorphism. Epidemiology.

[22] Yavuz E., Oktem M., Esinler I., Toru S.A., Zeyneloglu H.B. (2007). Genistein causes regression of endometriotic implants in the rat model. Fertil. Steril..

[23] Laschke M.W., Schwender C., Scheuer C., Vollmar B., Menger M.D. (2008). Epigallocatechin-3-gallate inhibits estrogen-induced activation of endometrial cells in vitro and causes regression of endometriotic lesions in vivo. Hum. Reprod..

[24] Xu H., Lui W.T., Chu C.Y., Ng P.S., Wang C.C., Rogers M.S. (2009). Anti-angiogenic effects of green tea catechin on an experimental endometriosis mouse model. Hum. Reprod..

[25] Laschke M.W., Schwender C., Vollmar B., Menger M.D. (2010). Genistein does not affect vascularization and blood perfusion of endometriotic lesions and ovarian follicles in dorsal skinfold chambers of Syrian golden hamsters. Reprod. Sci..

[26] Bruner-Tran K.L., Osteen K.G., Taylor H.S., Sokalska A., Haines K., Duleba A.J. (2011). Resveratrol inhibits development of experimental endometriosis in vivo and reduces endometrial stromal cell invasiveness in vitro. Biol. Reprod..

[27] Chen Y., Chen C., Shi S., Han J., Wang J., Hu J., Liu Y., Cai Z., Yu C. (2011). Endometriotic implants regress in rat models treated with puerarin by decreasing estradiol level. Reprod. Sci..

[28] Wang D., Liu Y., Han J., Zai D., Ji M., Cheng W., Xu L., Yang L., He M., Ni J. (2011). Puerarin suppresses invasion and vascularization of endometriosis tissue stimulated by 17β-estradiol. PLoS ONE.

[29] Xu H., Becker C.M., Lui W.T., Chu C.Y., Davis T.N., Kung A.L., Birsner A.E., D’Amato R.J., Wai Man G.C., Wang C.C. (2011). Green tea epigallocatechin-3-gallate inhibits angiogenesis and suppresses vascular endothelial growth factor C/vascular endothelial growth factor receptor 2 expression and signaling in experimental endometriosis in vivo. Fertil. Steril..

[30] Cheng W., Chen L., Yang S., Han J., Zhai D., Ni J., Yu C., Cai Z. (2012). Puerarin suppresses proliferation of endometriotic stromal cells partly via the MAPK signaling pathway induced by 17ß-estradiol-BSA. PLoS ONE.

[31] Maia H., Haddad C., Pinheiro N., Casoy J. (2012). Advantages of the association of resveratrol with oral contraceptives for management of endometriosis-related pain. Int. J. Womens Health.

[32] Rudzitis-Auth J., Körbel C., Scheuer C., Menger M.D., Laschke M.W. (2012). Xanthohumol inhibits growth and vascularization of developing endometriotic lesions. Hum. Reprod..

[33] Ergenoglu A.M., Yeniel A.O., Erbas O., Aktug H., Yildirim N., Ulukus M., Taskiran D. (2013). Regression of endometrial implants by resveratrol in an experimentally induced endometriosis model in rats. Reprod. Sci..

[34] Ji M., Liu Y., Yang S., Zhai D., Zhang D., Bai L., Wang Z., Yu J., Yu C., Cai Z. (2013). Puerarin suppresses proliferation of endometriotic stromal cells in part via differential recruitment of nuclear receptor coregulators to estrogen receptor-α. J. Steroid Biochem. Mol. Biol..

[35] Ricci A.G., Olivares C.N., Bilotas M.A., Bastón J.I., Singla J.J., Meresman G.F., Barañao R.I. (2013). Natural therapies assessment for the treatment of endometriosis. Hum. Reprod..

[36] Rudzitis-Auth J., Menger M.D., Laschke M.W. (2013). Resveratrol is a potent inhibitor of vascularization and cell proliferation in experimental endometriosis. Hum. Reprod..

[37] Wang C.C., Xu H., Man G.C., Zhang T., Chu K.O., Chu C.Y., Cheng J.T., Li G., He Y.X., Qin L. (2013). Prodrug of green tea epigallocatechin-3-gallate (Pro-EGCG) as a potent anti-angiogenesis agent for endometriosis in mice. Angiogenesis.

[38] Amaya S.C., Savaris R.F., Filipovic C.J., Wise J.D., Hestermann E., Young S.L., Lessey B.A. (2014). Resveratrol and endometrium: A closer look at an active ingredient of red wine using in vivo and in vitro models. Reprod. Sci..

[39] Demirel M.A., Suntar I., Ilhan M., Keles H., Kupeli Akkol E. (2014). Experimental endometriosis remission in rats treated with Achillea biebersteinii Afan: Histopathological evaluation and determination of cytokine levels. Eur. J. Obstet. Gynecol. Reprod. Biol..

[40] Matsuzaki S., Darcha C. (2014). Antifibrotic properties of epigallocatechin-3-gallate in endometriosis. Hum. Reprod..

[41] Taguchi A., Wada-Hiraike O., Kawana K., Koga K., Yamashita A., Shirane A., Urata Y., Kozuma S., Osuga Y., Fujii T. (2014). Resveratrol suppresses inflammatory responses in endometrial stromal cells derived from endometriosis: A possible role of the sirtuin 1 pathway. J. Obstet. Gynaecol. Res..

[42] Yavuz S., Aydin N.E., Celik O., Yilmaz E., Ozerol E., Tanbek K. (2014). Resveratrol successfully treats experimental endometriosis through modulation of oxidative stress and lipid peroxidation. J. Cancer Res. Ther..

[43] Bayoglu Tekin Y., Guven S., Kirbas A., Kalkan Y., Tumkaya L., Guvendag Guven E.S. (2015). Is resveratrol a potential substitute for leuprolide acetate in experimental endometriosis?. Eur. J. Obstet. Gynecol. Reprod. Biol..

[44] Cenksoy O.P., Oktem M., Erdem O., Karakaya C., Cenksoy C., Erdem A., Guner H., Karabacak O. (2015). A potential novel treatment strategy: Inhibition of angiogenesis and inflammation by resveratrol for regression of endometriosis in an experimental rat model. Gynecol. Endocrinol..

[45] Singh A.K., Chakravarty B., Chaudhury K. (2015). Nanoparticle-assisted combinatorial therapy for effective treatment of endometriosis. J. Biomed. Nanotechnol..

[46] Di Paola R., Fusco R., Gugliandolo E., Crupi R., Evangelista M., Granese R., Cuzzocrea S. (2016). Co-micronized palmitoylethanolamide/polydatin treatment causes endometriotic lesion regression in a rodent model of surgically induced endometriosis. Front. Pharmacol..

[47] Taguchi A., Koga K., Kawana K., Makabe T., Sue F., Miyashita M., Yoshida M., Urata Y., Izumi G., Tkamura M. (2016). Resveratrol enhances apoptosis in endometriotic stromal cells. Am. J. Reprod. Immunol..

[48] Kim J.H., Woo J.H., Kim H.M., Oh M.S., Jang D.S., Choi J.H. (2017). Anti-endometriotic effects of pueraria flower extract in human endometriotic cells and mice. Nutrients.

[49] Mendes da Silva D., Gross L.A., Neto E.P.G., Lessey B.A., Savaris R.F. (2017). The use of resveratrol as an adjuvant treatment of pain in endometriosis: A randomized clinical trial. J. Endocr. Soc..

[50] Park S., Lim W., Bazer F.W., Song G. (2017). Naringenin induces mitochondria-mediated apoptosis and endoplasmic reticulum stress by regulating MAPK and AKT signal transduction pathways in endometriosis cells. Mol. Hum. Reprod..

[51] Ferella L., Bastón J.I., Bilotas M.A., Singla J.J., González A.M., Olivares C.N., Meresman G.F. (2018). Active compounds present inRosmarinus officinalis leaves andScutellaria baicalensis root evaluated as new therapeutic agents for endometriosis. Reprod. Biomed. Online.

[52] Jouhari S., Mohammadzadeh A., Soltanghoraee H., Mohammadi Z., Khazali S., Mirzadegan E., Lakpour N., Fatemi F., Zafardoust S., Mohazzab A. (2018). Effects of silymarin, cabergoline and letrozole on rat model of endometriosis. Taiwan J. Obstet. Gynecol..

[53] Melekoglu R., Ciftci O., Eraslan S., Cetin A., Basak N. (2018). The beneficial effects of nerolidol and hesperidin on surgically induced endometriosis in a rat model. Gynecol. Endocrinol..

[54] Nahari E., Razi M. (2018). Silymarin amplifies apoptosis in ectopic endometrial tissue in rats with endometriosis; implication on growth factor GDNF, ERK1/2 and Bcl-6b expression. Acta Histochem..

[55] Park S., Lim W., Bazer F.W., Song G. (2018). Apigenin induces ROS-dependent apoptosis and ER stress in human endometriosis cells. J. Cell Physiol..

[56] Signorile P.G., Viceconte R., Baldi A. (2018). Novel dietary supplement association reduces symptoms in endometriosis patients. J. Cell Physiol..

[57] Takaoka O., Mori T., Ito F., Okimura H., Kataoka H., Tanaka Y., Koshiba A., Kusuki I., Shigehiro S., Amami T. (2018). Daidzein-rich isoflavone aglycones inhibit cell growth and inflammation in endometriosis. J. Steroid. Biochem. Mol. Biol..

[58] Wei X., Shao X. (2018). Nobiletin alleviates endometriosis via down-regulating NF-κB activity in endometriosis mouse model. Biosci. Rep..

[59] Arablou T., Delbandi A.A., Khodaverdi S., Arefi S., Kolahdouz-Mohammadi R., Heidari S., Mohammadi T., Aryaeian N. (2019). Resveratrol reduces the expression of insulin-like growth factor-1 and hepatocyte growth factor in stromal cells of women with endometriosis compared with nonendometriotic women. Phytother. Res..

[60] Ding D., Cai X., Zheng H., Guo S.W., Liu X. (2019). Scutellarin suppresses platelet aggregation and stalls lesional progression in mouse with induced endometriosis. Reprod. Sci..

[61] Ham J., Kim J., Bazer F.W., Lim W., Song G. (2019). Silibinin-induced endoplasmic reticulum stress and mitochondrial dysfunction suppress growth of endometriotic lesions. J. Cell Physiol..

[62] Ilhan M., Ali Z., Khan I.A., Taştan H., Küpeli Akkol E. (2019). Bioactivity-guided isolation of flavonoids from Urtica dioica L. and their effect on endometriosis rat model. J. Ethnopharmacol..

[63] Ilhan M., Ali Z., Khan I.A., Tastan H., Kupeli Akkol E. (2019). Promising activity of Anthemis austriaca Jacq. on the endometriosis rat model and isolation of its active constituents. Saudi Pharm. J..

[64] Kapoor R., Sirohi V.K., Gupta K., Dwivedi A. (2019). Naringenin ameliorates progression of endometriosis by modulating Nrf2/Keap1/HO1 axis and inducing apoptosis in rats. J. Nutr. Biochem..

[65] Kodarahmian M., Amidi F., Moini A., Kashani L., Shabani Nashtaei M., Pazhohan A., Bahramrezai M., Berenjian S., Sobhani A. (2019). The modulating effects of Resveratrol on the expression of MMP-2 and MMP-9 in endometriosis women: A randomized exploratory trial. Gynecol. Endocrinol..

[66] Ryu S., Bazer F.W., Lim W., Song G. (2019). Chrysin leads to cell death in endometriosis by regulation of endoplasmic reticulum stress and cytosolic calcium level. J. Cell Physiol..

[67] Park S., Lim W., Song G. (2019). Delphinidin induces antiproliferation and apoptosis of endometrial cells by regulating cytosolic calcium levels and mitochondrial membrane potential depolarization. J. Cell Biochem..

[68] Park S., Lim W., Bazer F.W., Whang K.Y., Song G. (2019). Quercetin inhibits proliferation of endometriosis regulating cyclin D1 and its target microRNAs in vitro and in vivo. J. Nutr. Biochem..

[69] Park S., Lim W., You S., Song G. (2019). Ameliorative effects of luteolin against endometriosis progression in vitro and in vivo. J. Nutr. Biochem..

[70] Bina F., Daglia M., Santarcangelo C., Baeeri M., Abdollahi M., Nabavi S.M., Tabarrai M., Rahimi R. (2020). Phytochemical profiling and ameliorative effects of Achillea cretica L. on rat model of endometriosis. J. Ethnopharmacol..

[71] Hernandes C., de Oliveira R.N., de Souza Santos A.H., Malvezzi H., de Azevedo B.C., Gueuvoghlanian-Silva B.Y., Pereira A.M.S., Podgaec S. (2020). The Effect of rutin and extracts of uncaria guianensis (Aubl.) J. F. gmeland on primary endometriotic cells: A 2D and 3D study. Molecules.

[72] Hsu Y.W., Chen H.Y., Chiang Y.F., Chang L.C., Lin P.H., Hsia S.M. (2020). The effects of isoliquiritigenin on endometriosis in vivo and in vitro study. Phytomedicine.

[73] Ilhan M., Ali Z., Khan I.A., Taştan H., Küpeli Akkol E. (2020). The regression of endometriosis with glycosylated flavonoids isolated from Melilotus officinalis (L.) Pall. in an endometriosis rat model. Taiwan J Obstet. Gynecol..

[74] Khazaei M.R., Rashidi Z., Chobsaz F., Niromand E., Khazaei M. (2020). Inhibitory effect of resveratrol on the growth and angiogenesis of human endometrial tissue in an In Vitro three-dimensional model of endometriosis. Reprod. Biol..

[75] Park W., Park M.Y., Song G., Lim W. (2020). 5,7-Dimethoxyflavone induces apoptotic cell death in human endometriosis cell lines by activating the endoplasmic reticulum stress pathway. Phytother. Res..

[76] Park S., Song G., Lim W. (2020). Myricetin inhibits endometriosis growth through cyclin E1 down-regulation in vitro and in vivo. J. Nutr. Biochem..

[77] Youseflu S., Jahanian Sadatmahalleh S.H., Mottaghi A., Kazemnejad A. (2020). Dietary phytoestrogen intake and the risk of endometriosis in iranian women: A case-control study. Int. J. Fertil. Steril..

[78] Page M.J., McKenzie J.E., Bossuyt P.M., Boutron I., Hoffmann T.C., Mulrow C.D., Shamseer L., Tetzlaff J.M., Akl E.A., Brennan S.E. (2021). The PRISMA 2020 statement: An updated guideline for reporting systematic reviews. BMJ.

[79] Benford D., Halldorsson T., Jeger M.J., Knutsen H.K., More S., Naegeli H., Noteborn H., Ockleford C., Ricci A., EFSA Scientific Committee (2018). The principles and methods behind EFSA’s guidance on uncertainty analysis in scientific assessment. EFSA J..

[80] Agarwal S.K., Daniels A., Drosman S.R., Udoff L., Foster W.G., Pike M.C., Spicer D.V., Daniels J.R. (2015). Treatment of endometriosis with the GnRHa deslorelin and add-back estradiol and supplementary testosterone. Biomed. Res. Int..

[81] Abu Hashim H. (2014). Potential role of aromatase inhibitors in the treatment of endometriosis. Int. J. Womens Health.

[82] Nawathe A., Patwardhan S., Yates D., Harrison G.R., Khan K.S. (2008). Systematic review of the effects of aromatase inhibitors on pain associated with endometriosis. BJOG.

[83] Cos P., De Bruyne T., Apers S., Berghe D.V., Pieters L., Vlietinck A.J. (2003). Phytoestrogens: Recent developments. Planta Med..

[84] Wang T.-Y., Li Q., Bi K. (2018). Bioactive flavonoids in medicinal plants: Structure, activity and biological fate. Asian J. Pharm. Sci..

[85] Panche A.N., Diwan A.D., Chandra S.R. (2016). Flavonoids: An overview. J. Nutr. Sci..

[86] Graf B.A., Milbury P.E., Blumberg J.B. (2005). Flavonols, flavones, flavanones and humanhealth: Epidemiological evidence. J. Med. Food.

[87] Chan K.K.L., Siu M.K.Y., Jiang Y.-X., Wang J.-J., Leung T.H.Y., Ngan H.Y.S. (2018). Estrogen receptor modulators genistein, daidzein and ERB-041 inhibit cell migration, invasion, proliferation and sphere formation via modulation of FAK and PI3K/AKT signaling in ovarian cancer. Cancer Cell Int..

[88] Kuiper G.G.J.M., Carlsson B., Grandien K., Enmark E., Häggblad J., Nilsson S., Gustafsson J.-A. (1997). Comparison of the ligand binding specificity and transcript tissue distribution of estrogen receptors and β. Endocrinology.

[89] Konar N. (2013). Non-isoflavone phytoestrogenic compound contents of various legumes. Eur. Food Res. Technol..

[90] Onzalez-Mejia M.E., Voss O.H., Murnan E.J., Dose A.I. (2010). Apigenin-induced apoptosis of leukemia cells is mediated by a bimodal and differentially regulated residue-specific phosphorylation of heat-shock protein–27. Cell Death Dis..

[91] Cos P., Ying L., Calomme M., Hu J.P., Cimanga K., Van Poel B., Pieters L., Vlietinck A.A.J., Berghe D.V. (1998). Structure–activity relationship and classification of flavonoids as inhibitors of xanthine oxidase and superoxide scavengers. J. Nat. Prod..

[92] Shay J., Elbaz H.A., Lee I., Zielske S.P., Malek M.H., Hüttemann M. (2015). Molecular mechanisms and therapeutic effects of (-) -epicatechin and other polyphenols in cancer, inflammation, diabetes and neurodegeneration. Oxidative Med. Cell. Longev..

[93] Yang C.S., Wang H. (2016). Cancer preventive activities of tea catechins. Molecules.

[94] Salehi B., Fokou P.V.T., Sharifi-Rad M., Zucca P., Pezzani R., Martins N., Sharifi-Rad J. (2019). The Therapeutic potential of naringenin: A Review of clinical trials. Pharmaceuticals.

[95] Durazzo A., Lucarini M., Camilli E., Marconi S., Gabrielli P., Lisciani S., Gambelli L., Aguzzi A., Novellino E., Santini A. (2018). Dietary lignans: Definition, description and research trends in databases development. Molecules.

[96] Cotterchio M., Boucher B., Kreiger N., Mills C.A., Thompson L.U. (2007). Dietary phytoestrogen intake—Lignans and isoflavones—And breast cancer risk (Canada). Cancer Causes Control.

[97] Xiao Q., Zhu W., Feng W., Lee S.S., Leung A.W., Shen J., Gao L., Xu C. (2019). A review of resveratrol as a potent chemoprotective and synergistic agent in cancer chemotherapy. Front. Pharmacol..

[98] Sirerol J.A., Rodríguez M.L., Mena S., Asensi M.A., Estrela J.M., Ortega A.L. (2015). Role of natural stilbenes in the prevention of cancer. Oxidative Med. Cell. Longev..

[99] Berman A.Y., Motechin R.A., Wiesenfeld M.Y., Holz M.K. (2017). The therapeutic potential of resveratrol: A review of clinical trials. NPJ Precis. Oncol..

[100] Nawaz W., Zhou Z., Deng S., Ma X., Ma X., Li C., Shu X. (2017). Therapeutic versatility of resveratrol derivatives. Nutrients.

[101] Ko J.-H., Sethi G., Um J.-Y., Shanmugam M.K., Arfuso F., Kumar A.P., Bishayee A., Ahn K.S. (2017). The role of resveratrol in cancer therapy. Int. J. Mol. Sci..

[102] Dinsdale N., Nepomnaschy P., Crespi B. (2021). The evolutionary biology of endometriosis. Evol. Med. Public Health.

