# Supplementary material for: Endometriosis and Phytoestrogens: Friends or Foes? A Systematic Review
Source: Nutrients. 2021 Jul 24;13(8):2532. doi: 10.3390/nu13082532 (PMC8398277; doi:10.3390/nu13082532)
Supplement: Supplementary file 1 [file nutrients-13-02532-s001.zip › nutrients-1282705-supplementary.pdf]

Supplementary Figure S1: Risk of bias assessment according to the risk of bias tool by Clarity Group [79].

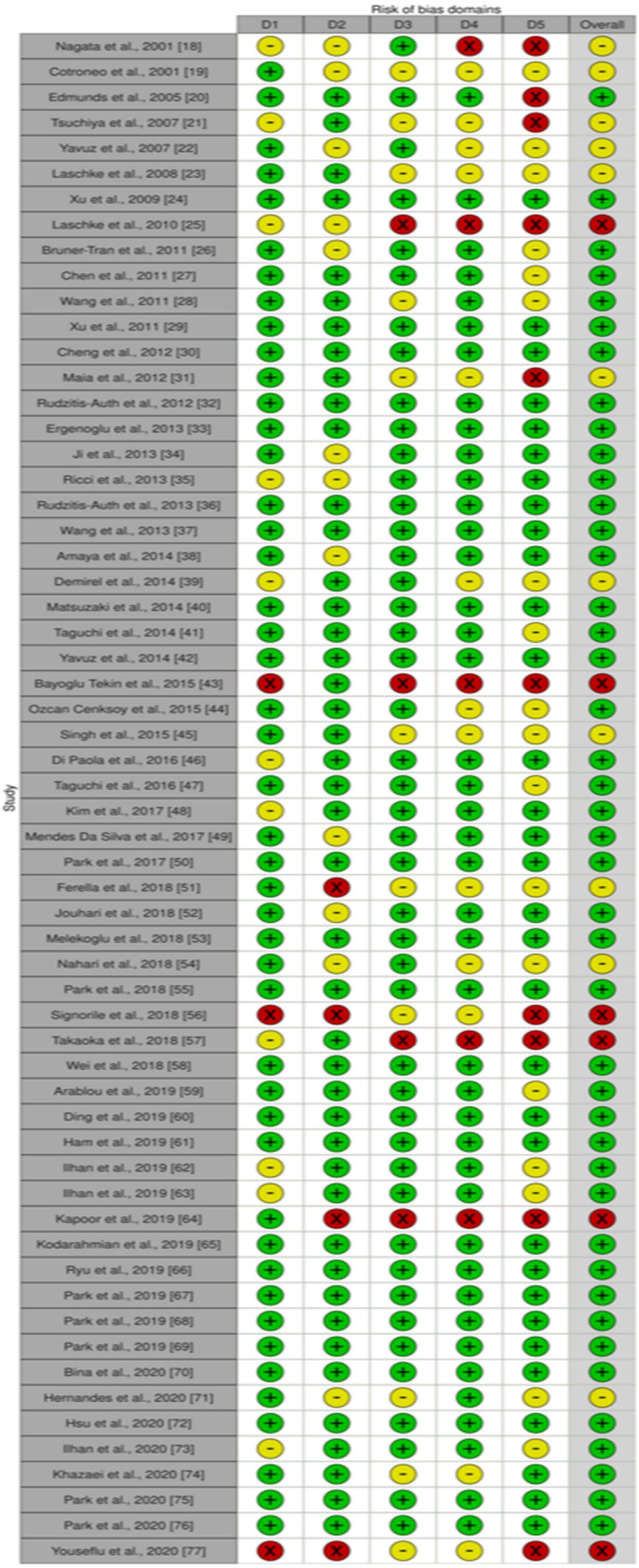

Legend: The risk of bias was assigned according to the following domains: D1) Can we be confident in the assessment of exposure?; D2) Can we be confident that cases had developed the outcome of interest and controls had not?; D3)Were the cases properly selected?; D4)Were the controls properly selected?; D5) Were cases and controls matched according to important prognostic variables or was statistical adjustment carried out for those variables?. We graded each potential source of bias as Definitely yes (low risk of bias), Probably yes (Moderate risk of bias), Probably no (Serious risk of bias), or Definitely no (Critical, high risk of bias).
